# An effective up-sampling approach for breast cancer prediction with imbalanced data: A machine learning model-based comparative analysis

**DOI:** 10.1371/journal.pone.0269135

**Published:** 2022-05-27

**Authors:** Tuan Tran, Uyen Le, Yihui Shi

**Affiliations:** 1 College of Pharmacy, California Northstate University, Elk Grove, CA, United States of America; 2 College of Medicine, California Northstate University, Elk Grove, CA, United States of America; Hanyang University, KOREA, REPUBLIC OF

## Abstract

Early detection of breast cancer plays a critical role in successful treatment that saves thousands of lives of patients every year. Despite massive clinical data have been collected and stored by healthcare organizations, only a small portion of the data has been used to support decision-making for treatments. In this study, we proposed an engineered up-sampling method (ENUS) for handling imbalanced data to improve predictive performance of machine learning models. Our experiment results showed that when the ratio of the minority to the majority class is less than 20%, training models with ENUS improved the balanced accuracy 3.74%, sensitivity 8.36% and F1 score 3.83%. Our study also identified that XGBoost Tree (*XGBTree*) using ENUS achieved the best performance with an average balanced accuracy of 97.47% (min = 93%, max = 100%), sensitivity of 97.88% (min = 89% and max = 100%), and F1 score of 96.20% (min = 89.5%, max = 100%) in the validation dataset. Furthermore, our ensemble algorithm identified *Cell_Shape* and *Nuclei* as the most important attributes in predicting breast cancer. The finding re-affirms the previous knowledge of the relationship between *Cell_Shape*, *Nuclei*, and the grades of breast cancer using a data-driven approach. Finally, our experiment showed that Random Forest and Neural Network models had the least training time. Our study provided a comprehensive comparison of a wide range of machine learning methods in predicting breast cancer risk. It can be used as a tool for healthcare practitioners to effectively detect and treat breast cancer.

## Introduction

### Overview

Over the past decades, healthcare information such as medical records and clinical data has been collected and stored in electronic databases. Both the governments and public organizations have accelerated the technology toward transparency by making massively stored data usable, searchable, and actionable [1]. Despite the massive healthcare databases available, only a small part of the data has been used by domain experts for diagnosis and treatments due to the complex and voluminous health data [2].

Breast cancer (BC) became the most commonly diagnosed cancer, overtaking lung cancer in 2020 [3]. In the United States, 287,850 new cases were diagnosed and 43,250 deaths were reported in female in 2022. It had the highest deaths among women aged 20 to 59 years [4]. In general, breast cancer tumors are classified into benign and malignant types. Although benign tumors are not life-threatening and cancerous, it may boost the chances of breast cancer risk. On the other hand, malignant tumors are cancerous and more alarming. Tremendous progress has been made in the treatment options for breast cancer patients in the last decade; however, one in eight men and one in eleven women still die from breast cancer. Early prediction of breast cancer is critical for successful treatment [5, 6]. However, the conventional approaches are limited in providing such a capability.

The recent breakthrough in machine learning (ML) and data mining (DM) techniques has provided new tools for healthcare practitioners in diagnostic and treatment [7–9]. A ML-based model can reduce the diagnosis errors and enhance the efficiency of cancer diagnosis by learning hidden information from massive training data that is difficulty to analyze directly. The applications of ML-based models have been successfully applied for predicting various diseases, such as lung cancer [10], heart disease [11], and thyroid cancer [12]. Although ML-based models performed well in various applications, the performance of a model will significantly depend on the characteristics of the features of the dataset as well as the techniques used to construct the model.

In this paper, we proposed a new up-sampling approach to improve the predictive performance of machine learning models. In addition, we analyzed the predictive power of the features across different models to identify predictor importance. Finally, we analyzed the runtime to compare the training effectiveness of different models. The experiment results showed that the proposed ENUS significantly improved the predictive performance of the models. We used the open-source programming language R to build our models. Our study provides a promising tool for early detection of breast cancer for effective treatments.

### Background and related work

Data mining is the process of using computer programs to analyze massive data to reveal hidden patterns or knowledge [13]. It is an interdisciplinary science including statistics, machine learning, information retrieval, bioinformatics, computer science, etc. Applications of data mining have been applied in many areas such as health sciences [2], finance [14], and the military [15].

Typically, the data mining process is divided into different phases as described by Pete et al. [16] including business understanding, data exploration and preparation, predictive modeling, evaluation, and deployment. These phases are repeatedly tuned and refined until the outcome of the model satisfies pre-specified requirements (e.g., prediction accuracy). Regarding the techniques, data mining can be categorized into supervised learning and unsupervised learning techniques. In the supervised learning techniques, the system is trained with a labeled dataset where the labels of the interested variable are known (labeled). The system learns the data patterns and predicts the labels of the target variable (e.g., benign/malignant) of new records. Several approaches fall into this category including Classification and Regression Trees (CART), Logistic Regression, Linear Regression, Neural Networks, k-Nearest Neighbors (k-NN), etc. In contrast, in the unsupervised learning, the system knows nothing about the target variable. Instead, the system takes input data, analyzes, and reveals hidden patterns (e.g., groups of similar patients or customers) of the data rather than predicting value (e.g., class or value) of a specific target variable. This approach includes clustering, outlier detection, and association rules.

There are several studies that used machine learning methods for predicting breast cancer risk [17–20]. Stark et al. [17] studied machine learning models using the PLCO dataset [21] including demographics and personal health data for predicting breast cancer risk. In another work, Turkki et al. [18] studied machine learning models using images of tumour samples as inputs to predict breast cancer risk. Furthermore, Boeri et al. [20] used machine learning techniques to predict breast cancer using both general health information (e.g., age, body mass index (BMI), etc.) and genomic data (e.g., BRCA-1 or BRCA-2 mutation, etc.). The results showed that machine learning models can achieve a good prediction accuracy for breast cancer.

There are some similar studies to ours but using a different dataset from the Wisconsin Breast Cancer database [22–24]. Using a larger dataset with more features, these studies have shown the feasibility of machine learning based models in predicting breast cancer risks in the healthcare domains. Additionally, Awais et al. [25] studied a human-in-the-loop-system to support diagnosis using advanced machine learning approaches. The proposed approach used a textural patterns of oral mucosal lesions and oral potentially malignant disorders to differentiate them from normal regions of the oral cavity via autofluorescence imaging. The study achieved 83% accuracy, 85% sensitivity, and 84% specificity in differentiating between normal and anomalous regions of the oral cavity. Furthermore, Shah et al. proposed a smart cardiac framework for an early detection of cardiac arrest condition and risk in [26]. In this study, the authors collected real-tine data using sensors and stored it on a cloud platform. Different machine learning models including Artifical Neural Network (ANN), Random Forest Classifier (RFC), Gradient Boost Extreme Gradient Boosting (XGBoost) classifier, Support Vector Machine (SVM), Naive Bayes (NB), and Decision Tree (DT) have been developed for cardiac arrest risk detection. The authors showed that DT achieved the best performance with a prediction accuracy of 98%.

Distinct from the existing studies, our work considered a practical small-size dataset for predicting breast cancer risk using advanced machine learning models. Particularly, we proposed an effective method for handling imbalanced data via an engineered up-sampling method (ENUS). The proposed ENUS approach generates new examples to generalize the classification boundary of the minority class that helps to improve the prediction performance of the models. In addition, our ensemble algorithm was developed to identify important predictors of breast cancer. Furthermore, we also measured the effectiveness of different models by comparing their training times. Several performance measures have been reported to demonstrate the effectiveness and efficacy of our approach. Ours is one of a few studies that provides a comprehensive comparison of state-of-the-art machine learning models for predicting breast cancer using a small-size dataset.

Our study contributes to the existing knowledge in different aspects.

- We first demonstrated that machine learning techniques could be used to predict breast cancer risk with high prediction accuracy using a small-size dataset. Particularly, via data visualization and exploratory data analysis (EDA), we showed that the underlying relationship among the attributes and target variable could be identified. The extracted information can be utilized during training process to improve the predictive performance of the models.
- Next, we provided an effective pre-processing method via engineered up-sampling (ENUS) to overcome the issues of imbalanced data. The models trained with ENUS significantly improved the balanced accuracy by 2.4% and sensitivity by 4.46% compared to those trained with the original data. In the range of a smaller ratio between the minority to majority class (e.g., less than 20%), the improvement is larger with 3.74% balanced accuracy and 8.36% sensitivity.
- In addition, our study analyzed the underlying relationship between the attributes and target variable to identify the feature importance in predicting breast cancer. We provided answer to the important question in machine modeling: which attributes contributed the most (or are the key predictors) to breast cancer? Our study revealed that *Cell_Shape* and *Nuclei* are the most important predictors in classifying breast cancer risk. This is interesting result as it helps to identify the hidden links between the key predictors and breast cancer risk, and it helps healthcare practitioners develop clinical insights and identifies pathways contributing to disease progression. To the best of our knowledge, this is one of a few studies that identified feature importance for such a dataset.
- Finally, we quantified the training efficiency of different models by measuring the time needed to train each model. Several machine learning models have been implemented and compared in our study.

## Materials and methods

### Dataset

**Fig 1:**
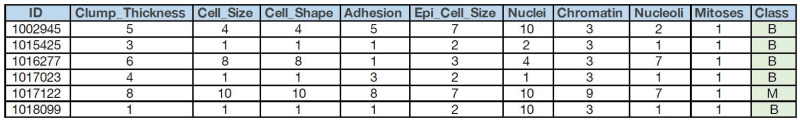
A sample of the dataset.

We used the well-known breast cancer dataset provided by the Wisconsin Breast Cancer Database [27]. The data attributes and a sample of the first six records in the dataset are shown in Table 1 and Fig 1, respectively.

**Table 1:** Data attributes.

No.	Attribute	Description	Value Range
1	*ID*	Sample code number	61634–13454352
2	*Clump*_*Thickness*	Clump Thickness	1–10
3	*Cell*_*Size*	Uniformity of Cell Size	1–10
4	*Cell*_*Shape*	Uniformity of Cell Shape	1–10
5	*Adhesion*	Marginal Adhesion	1–10
6	*Epi*_*Cell*_*Size*	Single Epithelial Cell Size	1–10
7	*Nuclei*	Bare Nuclei	1–10
8	*Chromatin*	Bland Chromatin	1–10
9	*Nucleoli*	Normal Nucleoli	1–10
10	*Mitoses*	Mitoses	1–10
11	*Class*	Target variable	B-Benign, M-Malignant

### Methods

#### Study design

**Fig 2:**
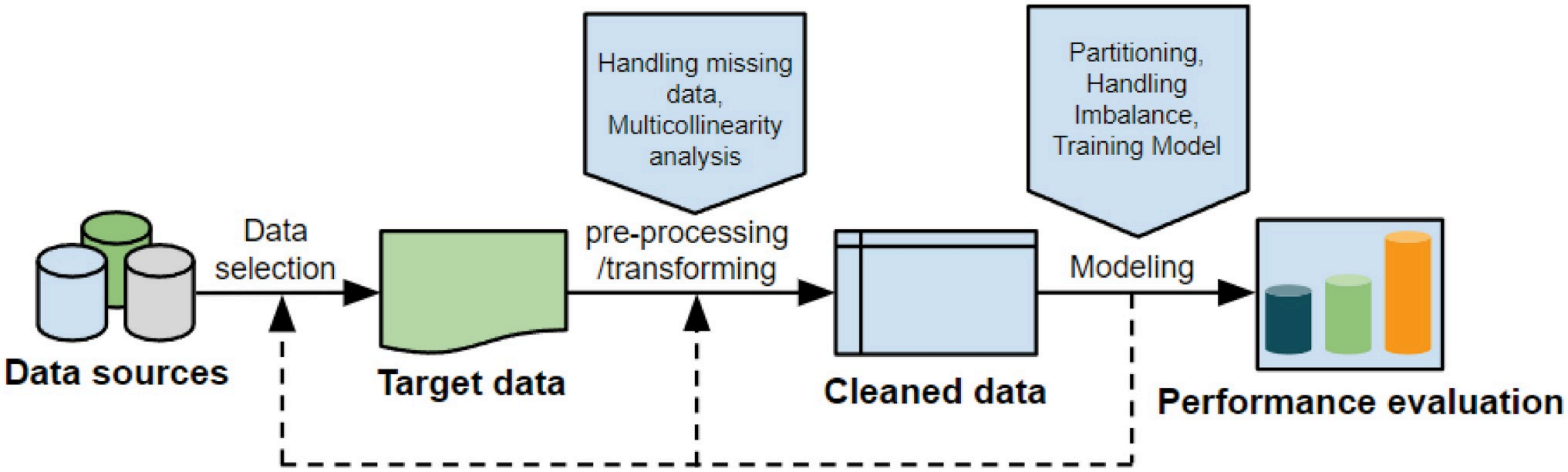
Modeling process overview.

Different from our previous study [28] where we used IBM SPSS Modeler 15 [29], in this study, we used the *R* programming language [30] to analyze and train our predictive models. By using *R*, we have more flexibility in data manipulation and modeling, resulting in higher prediction accuracy. Our modeling procedure is shown in Fig 2.

- **Data selection**: We used the well-known breast cancer dataset provided by the University of Wisconsin Hospitals [27]. This dataset was collected by Dr. William H. Wolberg from his clinical cases over three years [31]. The rationale for using this dataset is that it contained typical clinical information of the patients at the early stage of the disease. Constructing accurate predictive models with limited information is crucial for early detection and successful treatment.
- **Data pre-processing and transformation**: In this step, we perform exploratory data analysis for detecting outliers and missing values. We also conduct a correlation analysis to identify strongly correlated attributes to avoid multi-collinearity. In addition, transformed data in this step was used to build different models for comparison. More detailed analysis is described in the following subsection “Exploratory data analysis”.
- **Predictive model construction**: In this step, the processed data is partitioned into training and evaluation datasets which are used for constructing and evaluating our models, respectively. For training our models, several models are trained either with the proposed ENUS or original data. More detail of the ENUS approach is described in subsection “The proposed engineered up-sampling (ENUS) for handling data imbalance” below.

We note that machine learning modeling is an iterative process; thus, each step in Fig 2 is repeated several times before obtaining the final model.

#### Exploratory data analysis

Data exploration is a crucial step in building predictive machine learning models. We first perform exploratory data analysis for identifying missing values and outliers.

**Fig 3:**
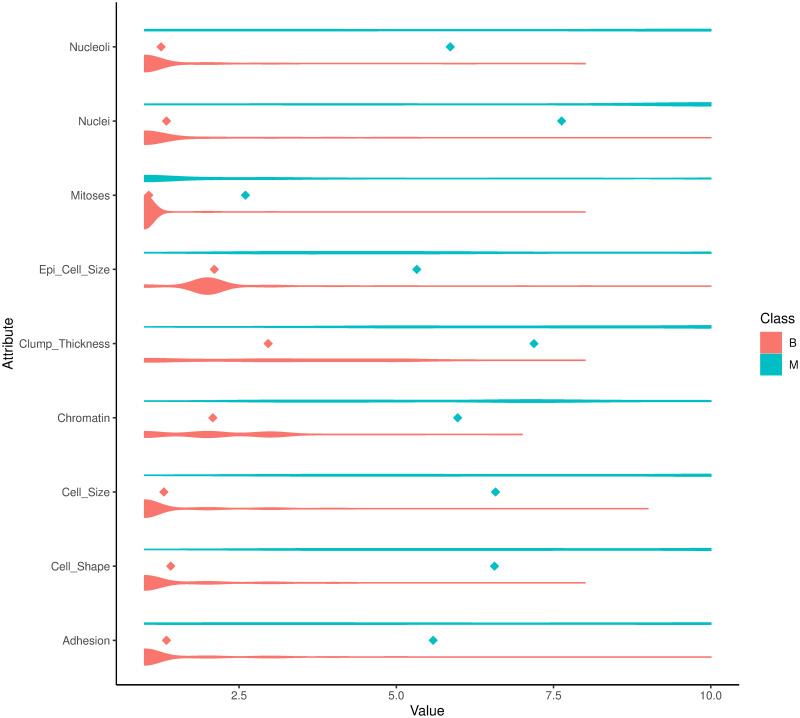
Distribution of the target “Class” attribute in the predictors.

**Fig 4:**
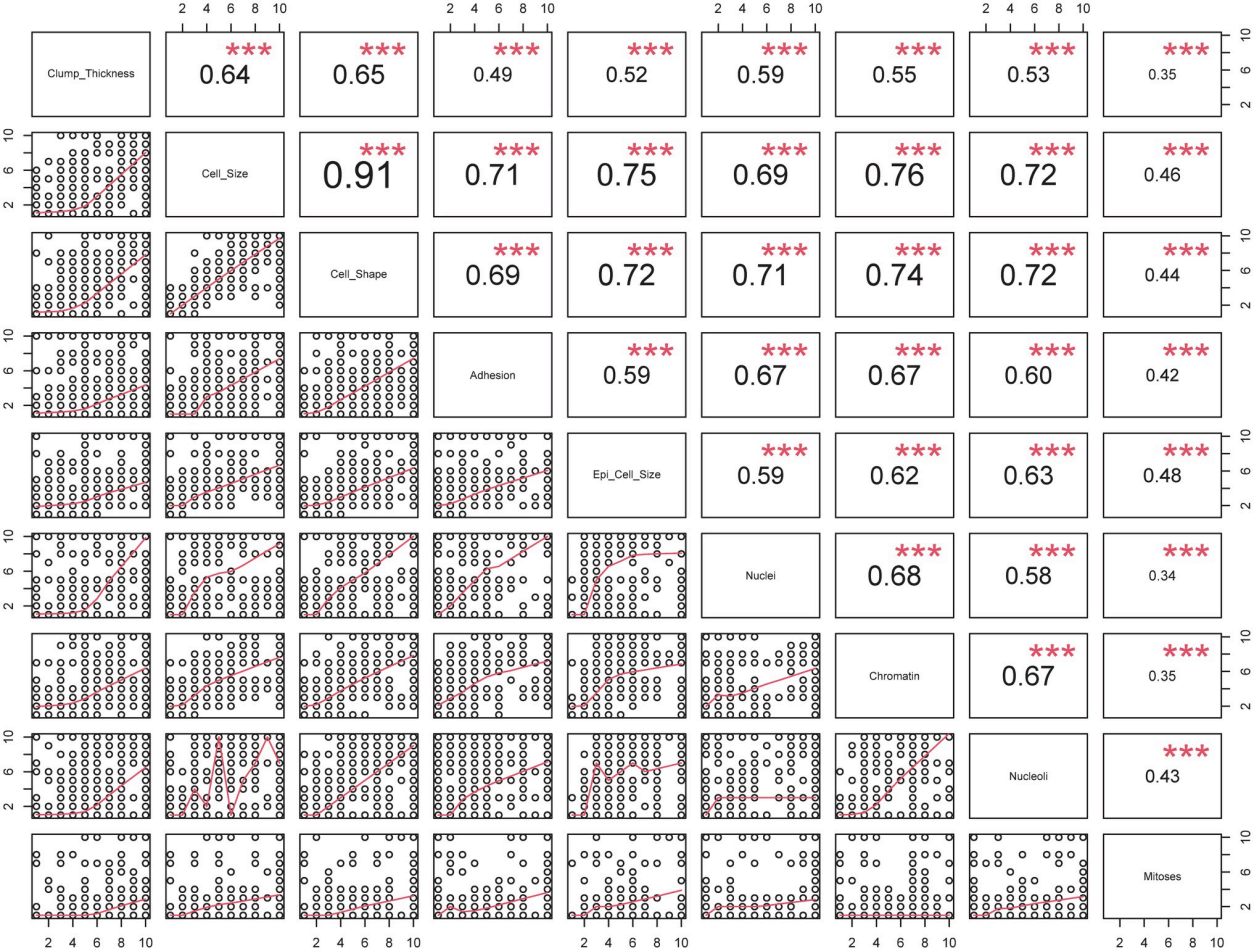
Correlation of predictors (*p*-value notation: “***” ≥ 0.001, “**” – (0.001, 0.01], “*” – (0.01, 0.05], “.” – (0.05, 0.1]).

**Fig 5:**
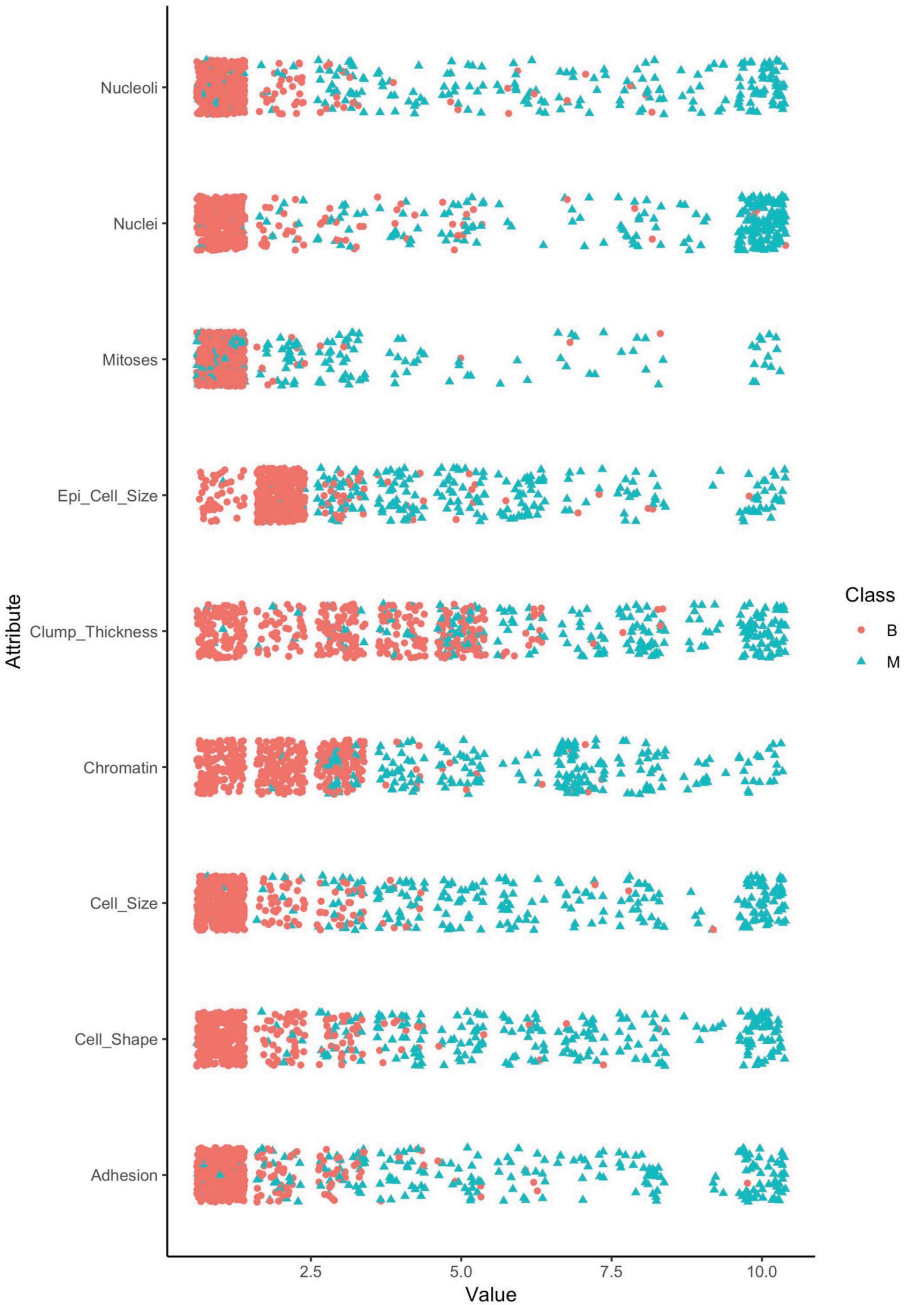
Distribution of class attribute in the predictors.

- **Identifying data size, outliers, and missing values**: In this step, we analyze the dataset and attributes to identify outliers and missing values.
  - *Dataset overview*: The original dataset has 698 observations and 11 variables including one identification field, nine clinical attributes and one class attribute.
  - *Missing values*: Our data exploratory analysis shows that “Nuclei” has 16 missing values which were encoded by “?”. We removed these observations resulting in 682 complete observations.
  - *Target class distribution*: Our analysis shows that there were 443 (64.96%) and 239 (35.04%) cases with the class of benign (B) and malignant (M), respectively. It is important to emphasize that the dataset was imbalanced and it should be taken into account when training the models. Particularly, we used the proposed ENUS approach to balance the benign and malignant cases in the training dataset for improving prediction performance.
  - *Distributions of attributes*: Next, we explore the distribution of the “Class” attribute versus the predictors in Fig 3. In this violin plot, the dots represent the mean values of the attributes classified by the target attribute. As we can see, the benign cases had smaller mean values compared to that of the malignant cases. We also observed that the *Cell*_*Shape* and *Cell_Size* have a similar distribution which implies a strong correlation of the two attributes. We further test this relationship in our next step.
- **Identifying attribute relationship**: Next, we analyze the relationships of the attributes by computing the Spearman correlation coefficients in Fig 4. We note that the Pearson correlation method should not be used in this case as our data is not normally distributed. The result confirmed our previous observation of the relationship between *Cell_Shape* and *Cell_Size* with a correlation coefficient of 0.91, *p*-value ≤ 0.001. We project that a model trained without using *Cell_Size* should achieve a similar performance compared to the one using it. In addition, we observe that all the features, except *Mitoses*, are moderate or strongly correlated with each other with correlation coefficients being greater than 0.49 (between *Clump_Thickness* and *Adhesion*). The weak correlations of *Mitoses* with other features indicate that *Mitoses* will not be an important predictor in classifying classes of the target variable.
- **Separation of classes in the target attribute**: Our goal here is to construct a model that uses predictors to correctly classify (i.e., separate) two classes “B” and “M” of the target attribute. As we showed in Fig 5, most of the attributes, the benign cases (B—denoted by red color) had small values (less than 2.5) whereas the malignant cases (M- denoted by green color) had larger values (greater than 3.0). We also observed that the distribution of the values of *Clump_Thickness* was evenly distributed between benign and malignant cases. This implies that this attribute may not be a good predictor in classifying the target variable.

#### The proposed engineered up-sampling (ENUS) for handling data imbalance

**Fig 6:**
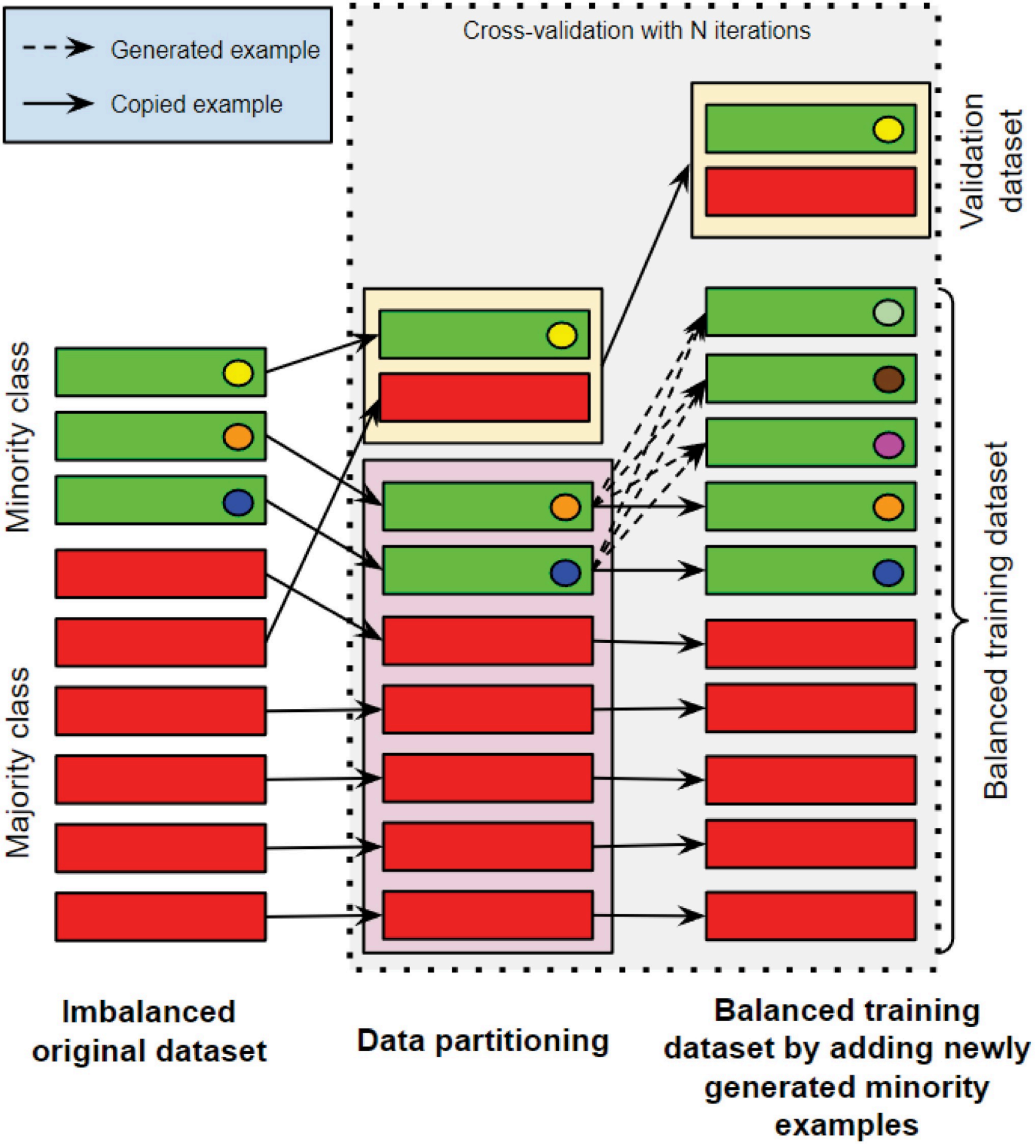
The proposed engineered up-sampling (ENUS) approach.

From our analysis above, the dataset is imbalanced where we had more benign cases (65%) than malignant cases (35%). We note that it is not an extreme case of imbalanced dataset but we show that by using ENUS we can improve the predictive performance of the models. Using ENUS, we construct new examples of the minority class by combining existing malignant cases in the training dataset. The proposed approach not only balances the training data to prevent the model from inclining towards the majority class, but also effectively shifts the decision boundaries of the minority class to be more general. The proposed up-sampling approach is illustrated in Fig 6.

For demonstration, in Fig 6, we assume that the dataset has three (3) and six (6) minority and majority examples, respectively. We first partition the original dataset into training and validation datasets. In our example, we assume that the validation and training datasets respectively have two (2) and seven (7) records as shown in Fig 6. The validation dataset will be set aside for evaluation while the training dataset is used to construct new examples of the minority class by combining the existing malignant cases (e.g., 2 cases in our example in Fig 6).

In ENUS, a new example is generated in the feature space of the existing minority examples. More specifically, to generate a new minority example, we randomly select a minority example (referred to as the Centroid) and its *k* nearest neighbors from the original training dataset. To construct the value of a feature for the new example, we multiply a random value between 0 and 1 with the difference of the distance between the Centroid and its nearest neighbor on that feature, and add that value to the value of Controid’s feature. We repeat the process to all other features of the training dataset to construct the values for all features of the new example. By using this process, we enforce our model to generalize its classification boundary beyond the existing examples of the original training dataset. As shown in Fig 6, the two original malignant examples are used to generate three new malignant examples. The balanced training dataset now has ten (10) examples in total, five examples for each class. This training dataset is then used to train our predictive models.

#### Modeling

In this subsection, we describe the steps used to construct predictive models for classifying patients into benign and malignant breast cancer categories.

*Data partitioning*. To construct our models, we randomly divide the dataset into two subsets, i.e., training and validation. The training and validation datasets contain 80% and 20% of the records, respectively. The training dataset is used for training the models whereas the validation dataset is held out and solely used for performance evaluation.

**Fig 7:**
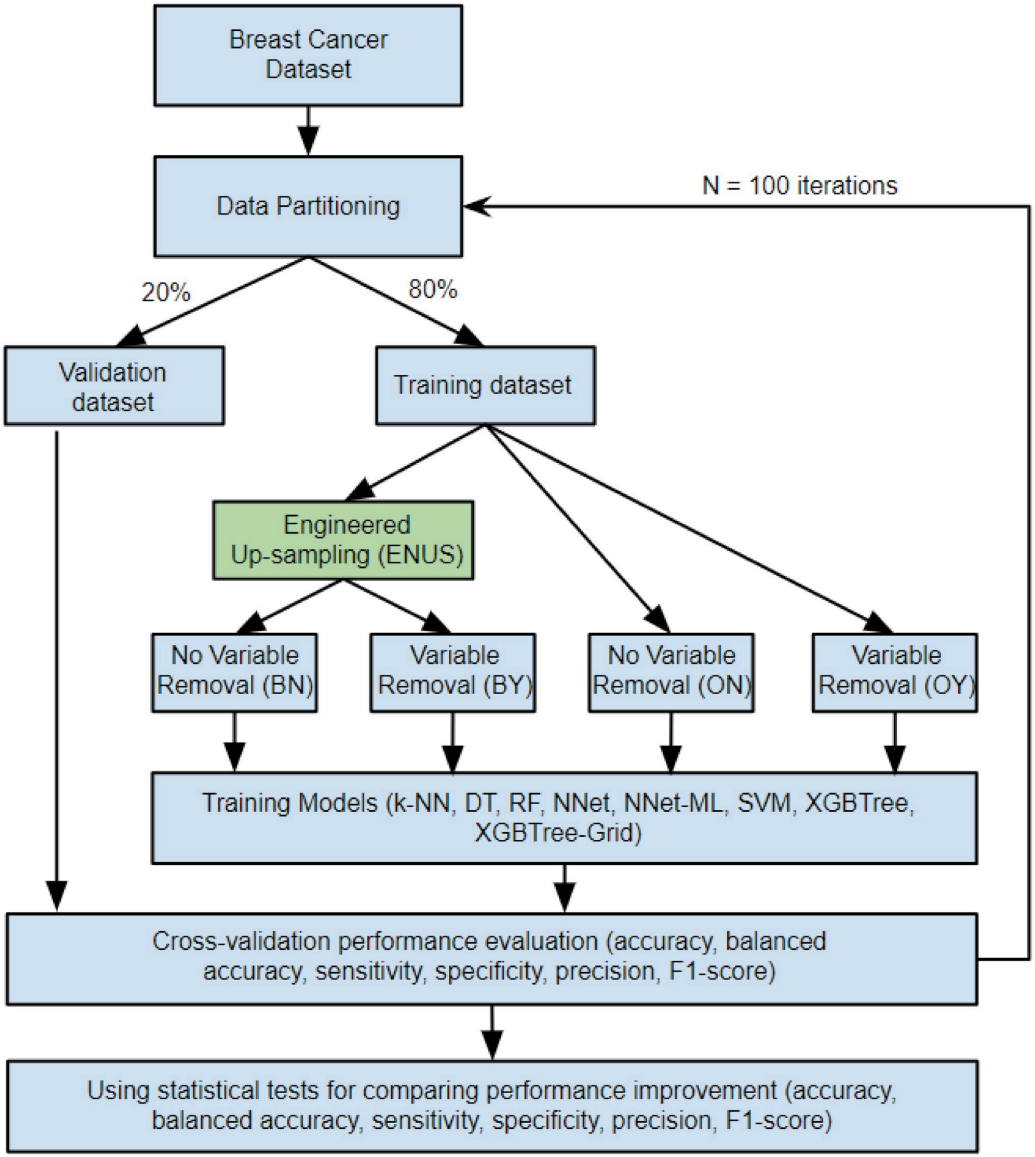
Experiment setup overview.

*Handling data imbalance*. Our models are either trained with original training data or ENUS-applied training data. When training with ENUS, the training data is up-sampled by using the proposed approach ENUS. Detail of the training process and experiment settings is described in subsection “Experiment setup” and Fig 7.

*Handling correlated variables*. Based on our exploratory data analysis, we identified two variables, *Cell_Size* and *Cell_Shape*, that are strongly correlated. Using both variables in constructing a predictive model may require more training time and could lower the predictive performance of the models due to multicollinearity [32]. For comparison, we train our models using both with and without *Cell_Size* feature.

*Designing models*. In this study, we implemented several machine learning models. The models, software packages, and hyperparameter tuning search algorithms are described in the next section.

- *k Nearest Neighbors (k-NN)*: We first started with the *k-NN* [33] model. This is an instance-based learning method where the label of a new patient is classified based on the voting of *k* nearest neighbors next to it. For instance, if *k = 1*, then the model is simply assigned to the class of that single nearest neighbor. On the other hand, if *k = n* (the size of the dataset), the model turns into a majority voting method. Our *k-NN* model is constructed based on the classification and regression software package (caret) [34]. In our implementation, the optimal value of *k* is achieved by searching in the range from 5 to 45 with 10-fold cross-validation on the training data.
- *Decision Tree (DT)*: We next construct a decision tree [35] using the *caret* package [34]. The implementation of the Recursive Partitioning and Regression Trees (rpart) model in *caret* allows us to use the build-in tree pruning function that avoids overfitting issues when constructing our classification tree. In our model, we used the complexity parameter (*cp*) to optimize our classification tree. Here the *cp* imposes a penalty on trees that have too many splits, potentially resulting in an overfitting tree. On the other hand, a larger value of *cp* would result in a too small tree. The optimal value of *cp* was estimated by testing different values of *cp* along with cross-validation to determine the best model with the highest prediction accuracy. Here we use 10-fold cross-validation and 10 possible values of *cp*.
- *Random Forest (RF)*: *RF* is an ensemble method that can be used to build predictive models for both classification and regression problems. The key concept of *RF* is to construct multiple trees using different subsets and features of the training data and then combine the results of these trees for final prediction. Intuitively, the *RF* model will perform better than a *DT* thanks to its ensemble results of several decision trees. To develop our *RF* model, we created an entire forest of random uncorrelated decision trees then combined their predicted outcomes. To construct the model, we used the Random Forest package [36]. Noting that the *randomForest()* function will generate errors if training data has missing values. In our study, we have handled the missing values in our “*Exploratory data analysis*” step. Some key parameters used when constructing *RF* model include:
  - *ntree*: This parameter defines the number of trees growing during the process. In our study, we set *ntree* = 500 [37] so that every observation gets predicted at least a few times to establish the error. However, we also want to limit the value of *ntree* because too large of a value may result in an overfitting model.
  - *mtry*: This parameter defines the number of variables randomly sampled as candidates at each split. The recommended value is around the squared root of the number of variables. We set *mtry = 3* in our model.
- *Neural Networks (NNet)*: Next, we use the *neuralnet* package to construct our neural network model [38]. *NNet* consists of an input layer, multiple hidden layers, and one output layer. The input layer takes the predictors as inputs and feeds these inputs to the next layers via weighted factors which were learned from the training dataset. The training process involves adjusting these parameters to accurately predict the class of the given inputs. In our study, we use the commonly used rectified linear unit (*ReLU*) as our activation function. The *NNet* used no hidden layer.
- *Multiple Layer Neural Networks (NNet-ML)*: This model used the same *neuralnet* package as in the *NNet* model. The only difference was that in the *NNet-ML* model, we use an additional hidden layer to construct the model. We expect that with an additional hidden layer, the *NNet-ML* will achieve a more robust performance compared to the *NNet* model.
- *Support Vector Machine (SVM)*: *SVM* is a data classification method that separates data using hyperplanes. The concept of *SVM* is to use hyperplanes to separate the data into different homogeneous data groups for classification. The *SVM* technique is generally useful for data that has non-regularity where the distribution of the data is unknown. In our study, we use two packages *caret* and *kernlab* to construct and tune the hyperparameters of the *SVM* model. Particularly, we implemented a grid search algorithm to identify an optimal set of the hyperparameters used in our final model. In our algorithm, we focused on two hyperparameters: 1) cost: control the error of the model; and 2) *γ*: control the curvature of the decision boundary of the Gaussian Radial Basis Function (RBF) kernel. The search ranges were based on earlier studies [39, 40]. The pseudocode of the algorithm is shown in Algorithm 1.
- *XGBoost Tree (XGBTree) and XGBTree-Grid Models*: XGBoost is short for the eXtreme Gradient Boosting algorithm, which can be used to construct models for both regression and classification [41]. Generally speaking, *XGBTree* is an ensemble decision tree algorithm where multiple decision trees are constructed and the outputs are combined to determine the final prediction. It is worth emphasizing that instead of combining the prediction outcomes at the end of the process as in the *RF* model, *XGBTree* combines the prediction outcomes of multiple decision trees along the way. This is to utilize the prediction power of multiple trees to improve the accuracy. Mathematically, the objective function of an *XGBTree* model can be expressed as [41]
  $$\begin{array}{c} L=\sum_{i=1}^{n}l\left(y_{i},{\hat{y}}_{i}\right)+\sum_{k=1}^{K}\Omega \left(f_{k}\right), \end{array}$$ (1)
  where *n* is the number of training examples, *y*_*i*_ and ${\hat{y}}_{i}$ are training labels and predicted labels, respectively; *K* is the number of trees, *f* is a function in the functional space *F* that includes all possible classification and regression trees (CARTs). Here the first and second parts of the equation represent the training loss and the regularization term, respectively. The training loss is the predictive accuracy of the model while the regularization controls the complexity of the model to avoid overfitting. When training our model, the goal is to balance these two parameters to construct a simple model with the highest prediction accuracy. In our modeling process, the dataframe format of the training data was transformed into a matrix form using the *as.matrix()* function to make our training data compatible with the input of the *xgboost()* function. We constructed two types of the *XGBTree* models for comparison purposes: i) a fixed set of the parameters (*XGBTree*), and ii) a grid-search to identify the optimal set of the hyperparameters (*XGBTree-Grid*).

**Algorithm 1: Grid search for optimal hyperparameters of SVM**.

**Result**: Optimal parameters for SVM model

initialization: *γ* = [0, 0.25], cost = [0.5, 8], kernel = {radial};

parms = expand.grid(cost = cost, gamma = *γ*, kernel = kernel);

**for** *i in 1:nrow(parms)* **do**

 model = svm(Class., data = trainb, gamma = parms$gamma[i], cost = parms$cost[i], kernel = parms$kernel[i]) #construct an svm with each set of the parameters;

 pre-svm = predict(model, training-data);

 predict = confusionMatrix(pre-svm, training-data$Class);

 acc[i] = predict$overall[1] #get overall accuracy;

**end**

acc = data.frame(p = seq(1,nrow(parms)), cnt = acc);

opt-p = subset(acc, cnt == max(cnt))[1,] #retrieve the optimal parameters

### Experiment setup

In our experiment, we divided the original dataset into training dataset (80%) and validation dataset (20%). The training dataset was used solely for training the models while the validation dataset was used to evaluate the performance of the models. For each model, four different configurations were used for training:

- ImBalanced pre-processing with No variable removal (BN): In this training configuration, training data were balanced by using the proposed ENUS to generate malignant data records to equalize the “M” and “B” cases in the target variable. All nine predictors were used to construct the models.
- ImBalanced pre-processing with variable removal (BY): In this training configuration, the training dataset was balanced by using ENSU and the *Cell_Size* variable was removed from the training dataset. Thus, only eight variables were used to construct the models.
- Original data with No variable removal (ON): In this training configuration, the original training dataset was used without balancing “B” and “M” cases in the target variable. All variables were used to construct the models.
- Original data with variable removal (OY): In this training configuration, the original training dataset was used without balancing the “B” and “M” classes and the *Cell_Size* variable was removed from the training dataset.

All the models were trained with 10-fold cross-validation and the best model was used for evaluation on the validation dataset. We conducted 100 trials and computed the average of the results for comparison. The overview of our experiment setup is illustrated in Fig 7 and optimal hyperparameters for the implemented models are in Table 2. We note that the optimal hyperparameters were obtained via grid search based on the default or recommended value ranges of the models [34, 38, 42].

**Table 2:** Optimal hyperparameters of the models.

Model	Optimal hyperparameters
*k* − *NN*	*k* = 20 (the number of nearest neighbors)
*DT*	*cp* = 0.01 (complexity parameter), tuneLength = 10 (grid tuning parameter)
*RF*	mtry = 3 (randomly selected predictors), tree_num = 500 (number of trees grown)
*NNet*	init_rand_weight = 0.5, *decay* = 5e-4, max_iteration = 5000, activation_func: *ReLU*
*NNet* − *ML*	hidden_layer_num = 8, max_step = 1e+5, learning_rate = 0.1, threshold = 0.01 (for partial derivatives of error func.), activation_func: *ReLU*.
*SVM*	method = *svmRadial*, *γ* = 0.1, *cost* = 1
*XGBTree*	learning_rate = 0.01, max_depth = 5, *γ* = 3 (min loss reduction).
*XGBTree* − *Grid*	learning_rate = 0.1, max_depth = 7, *γ* = 5 (min loss reduction).

## Results

### Performance metrics

To evaluate the performance of the proposed methods, we used several metrics which are recommended for evaluating a binary classifier [43] including prediction accuracy, balanced accuracy, sensitivity, specificity, precision, and F1-score. We emphasize that with imbalanced data, the balanced accuracy, sensitivity, and F1-score are important predictive performance measures when comparing different models.

- *Prediction Accuracy*: Prediction accuracy (*Acc*) is computed by the total numbers of cases (both benign and malignant) that were correctly classified divided by the total number of cases in the validation dataset. Mathematically, we have
  $$\begin{array}{c} Acc=\frac{TP+TN}{P+N}, \end{array}$$ (2)
  where *TP* and *TN* are the numbers of predicted true positive and true negative cases, respectively; *P* and *N* are the numbers of positive and negative cases of the validation dataset.
- *Balanced Prediction Accuracy*: In imbalanced dataset the *Acc* does not work well as most cases are benign; thus, the ratio of benign to malignant cases would be small. In this case, balanced accuracy (*bAcc*) is a good performance metric. *bAcc* is normalized by the true positive and true negative predictions.
  $$\begin{array}{c} bAcc=\frac{TPR+TNR}{2}, \end{array}$$ (3)
  where *TPR* and *TNR* are the true positive rate and true negative rate of the model.
- *Sensitivity and Specificity*: We also compare sensitivity (*Sen*) and specificity (*Spe*) of the models as well. Mathematically, we have the following formulas
  $$Sen=\frac{TP}{TP+FN}$$ (4)
  $$Spe=\frac{TN}{TN+FP},$$ (5)
  where *FN* and *FP* are the false negative and false positive cases, respectively.
- *Precision*: We also use precision (*Pre*) to compare our models. Here we have
  $$\begin{array}{c} Pre=\frac{TP}{TP+FP} \end{array}$$ (6)
- *F1-score*: Finally, we use the F1-score for comparing the performance of the models. F1-score is considered as the harmonic mean of the model as it is a combination of the model’s precision and sensitivity. Mathematically, we have
  $$\begin{array}{c} F1-score=\frac{2*Pre*Sen}{Pre+Sen}, \end{array}$$ (7)
  where *Sen* and *Pre* are the Sensitivity and Precision that are calculated in equations Eqs (4) and (6), respectively.

### Overall performance results

We conducted 100 runs in our experiment. For each run, we randomly selected 80% of the data for training the models and the remaining 20% of the data were used for evaluation. The average performance of each model by different metrics is shown in Table 3. We observed that *XGBTree* was the best model, with an average of 97.14% prediction accuracy (*Acc*) and 97.15% balanced accuracy (*bAcc*). On the contrary, *DT* had the worst performance with less than 94.2% of prediction accuracy. In addition, we observed that *NNet-ML* achieved higher performance compared to the *NNet* thanks to the additional hidden layer added to the model providing more flexibility in fitting the data. Interestingly, the data-driven model *k-NN* achieved a similar performance compared to the XGBTree on specificity (*Spe*) and precision (*Pre*) with an average of 97.20% and 94.9%, respectively. Finally, *XGBTree* achieved the highest performance on F1-score of 95.90%.

**Table 3:** Average prediction performance of the models.

Model	Acc	bAcc	Sen	Spe	Pre	F1-score
*k* − *NN*	96.5%	96.5%	96.3%	**97.2%**	**94.9%**	95.5%
*DT*	94.2%	93.9%	92.7%	95.1%	91%	91.7%
*RF*	96.6%	96.6%	96.8%	96.9%	94.3%	95.4%
*NNet*	95.3%	94.9%	93.2%	95.9%	92.5%	93.2%
*NNet* − *ML*	95.8%	95.5%	94.6%	96.4%	93.4%	93.9%
*SVM*	96.7%	96.6%	96.6%	96.8%	93.3%	95.3%
*XGBTree*	**97.14%**	**97.15%**	**97.20%**	97.2%	94.8%	**95.9%**
*XGBTree* − *Grid*	97%	97%	96.7%	97.2%	94.8%	95.7%

### Performance improvement significance statistical test

To evaluate the significance of performance improvement, we used the independent *t*-test to test the significance of differences of the average results across different models. Table 4 shows the *p*-value of the independent *t*-test between the performance of *XGBTree* and other models, with a 95% confidence interval. We observed that the performance improvement of the *XGBTree* compared to the other models was statistically significant, except *XGBTree-Grid* model. This is expected as *XGBTree-Grid* implemented the same classification model with additional grid algorithm that required a much longer training time compared to that of *XGBTree*. Detail of the evaluation of the training time will be discussed in the next subsection.

**Table 4:** *p*-value of the independent *t*-test comparing the performance of *XGBTree* with other models using a 95% confidence interval (Note: (*) implies the *p*-value is much smaller than 0.001).

Model	Acc	bAcc	Sen	Spe	Pre	F1-score
*k* − *NN*	0.012	(*)	(*)	0.7	0.77	0.07
*DT*	(*)	(*)	(*)	(*)	(*)	(*)
*RF*	0.011	(*)	0.01	0.04	0.054	0.05
*NNet*	(*)	(*)	(*)	(*)	(*)	(*)
*NNet* − *ML*	(*)	(*)	(*)	(*)	(*)	(*)
*SVM*	(*)	(*)	(*)	(*)	(*)	(*)
*XGBTree* − *Grid*	0.35	0.27	0.01	0.94	0.91	0.35

### Accuracy and balanced accuracy

**Fig 8:**
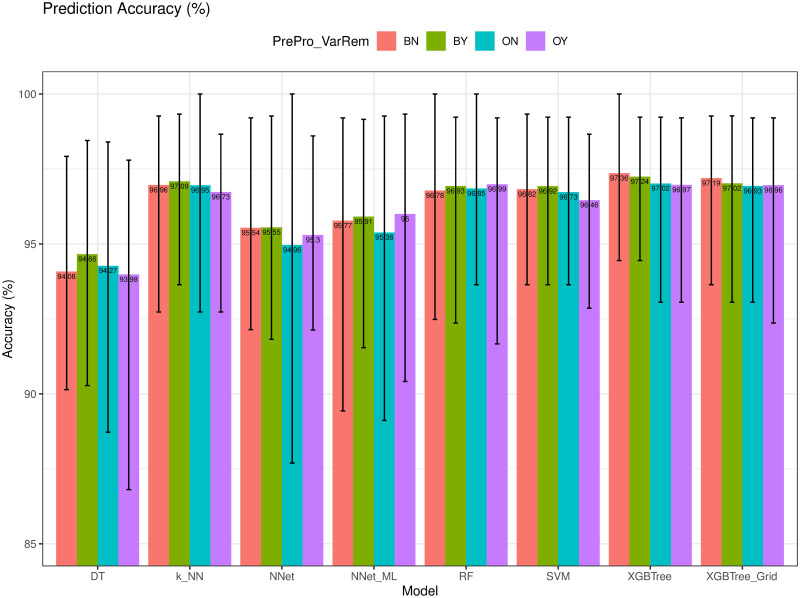
Prediction accuracy (*Acc*) of different models.

**Fig 9:**
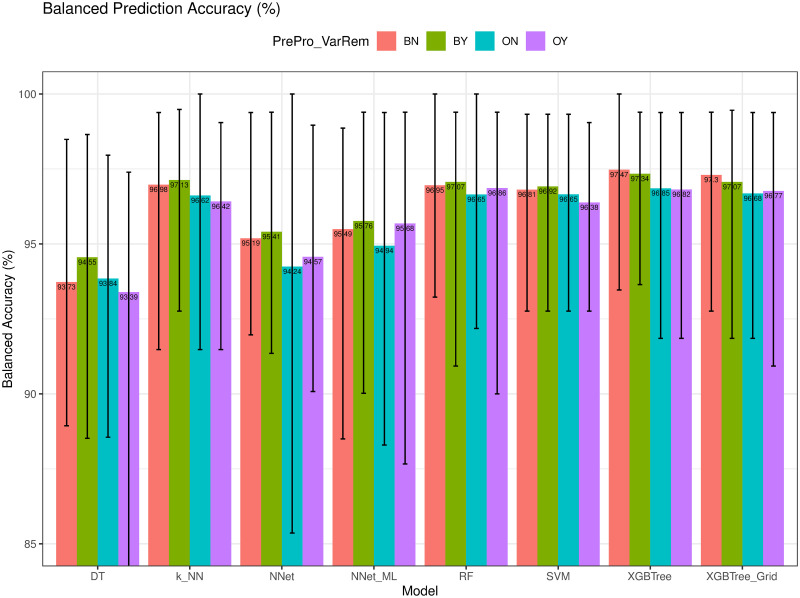
Balanced accuracy (*bAcc*) of different models.

We next compare the prediction accuracy (*Acc*) and Balanced Accuracy (*bAcc*) of the models using different training configurations (i.e., BN, BY, ON, and OY), as depicted in Figs 8 and 9, respectively. As we observed, XGBTree achieved the best with an average *Acc* = 97.36% (min = 94.40%, max = 100%), and *bAcc* = 97.47% (min = 93.50%, max = 100%). *DT* had the worst performance with an average *Acc* = 93.98% and *bAcc* = 93.39%. As we expected, the models using the proposed ENUS up-sampling method achieved much higher performance in predicting the class of breast cancer compared to the same models that did not. In addition, we observed that the removal of *Cell_Size* variable did improve the prediction accuracy on some models but not all.

### Sensitivity and specificity evaluation

**Fig 10:**
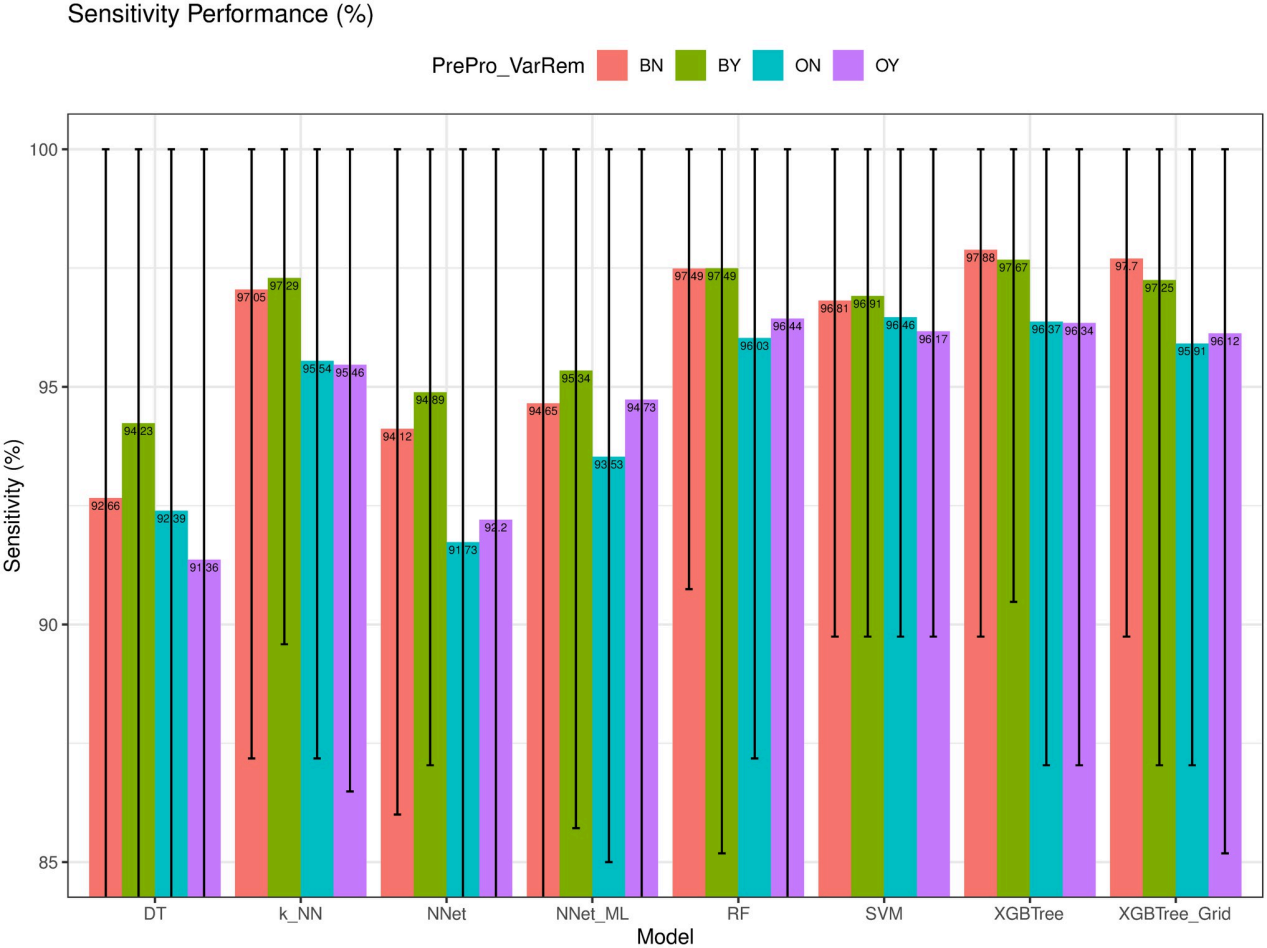
Sensitivity (*Sen*) of different models.

**Fig 11:**
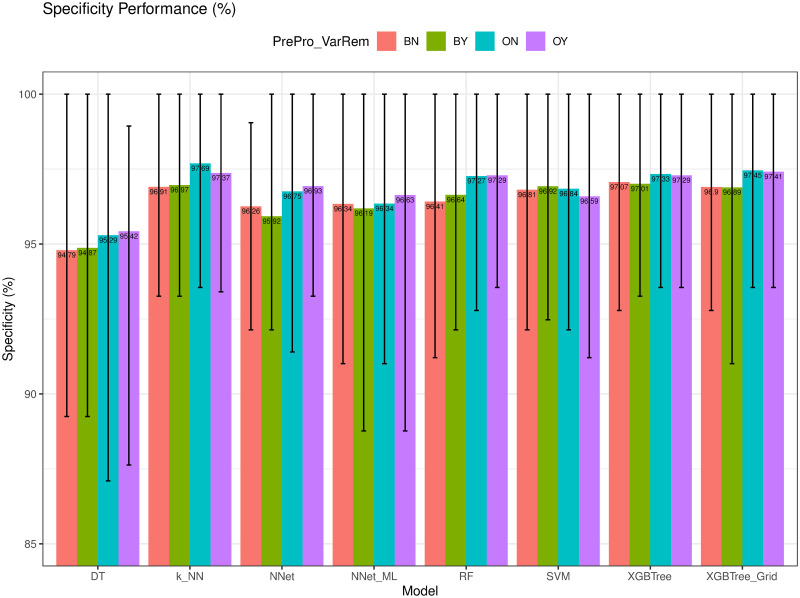
Specificity (*Spe*) of different models.

Next, we compare the sensitivity (*Sen*) and specificity (*Spe*) performances of different models in Figs 10 and 11. We note that *Sen* is the ability to detect a breast cancer when the patient has the disease whereas *Spe* is the ability to detect no breast cancer when the patient has no disease. We emphasize that, in this case, breast cancer is the minority class where the number of cases is much lower than that of the non-cancer class. *XGBTree* achieved the highest Sensitivity (97.88%) while *k-NN* had the highest Specificity (97.69%). *DT* had the lowest *Sen* = 91.36% and lowest *Spe* = 94.79%. The results showed that using ENUS in the training process significantly improves the ability of the models to correctly identify patients with the disease which is the minority class in this evaluation.

### Precision and F1-score evaluation

**Fig 12:**
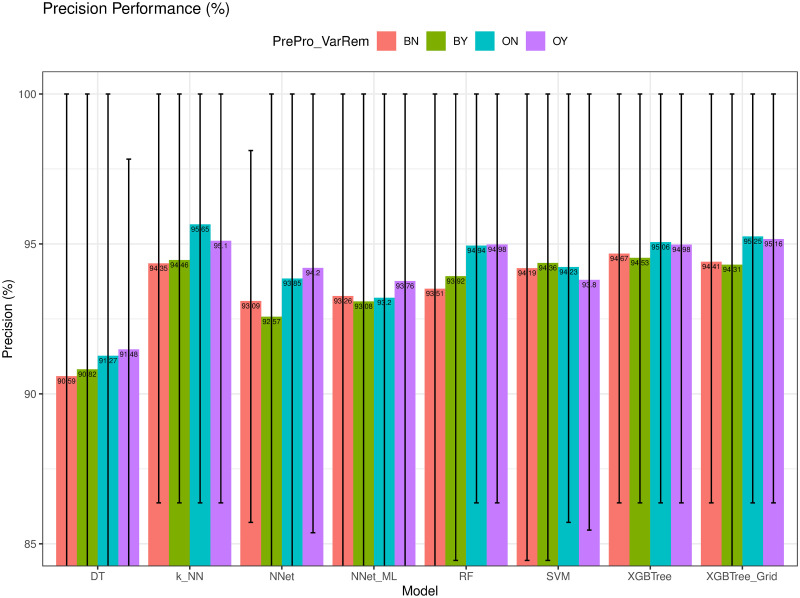
Precision (*Pre*) of different models.

**Fig 13:**
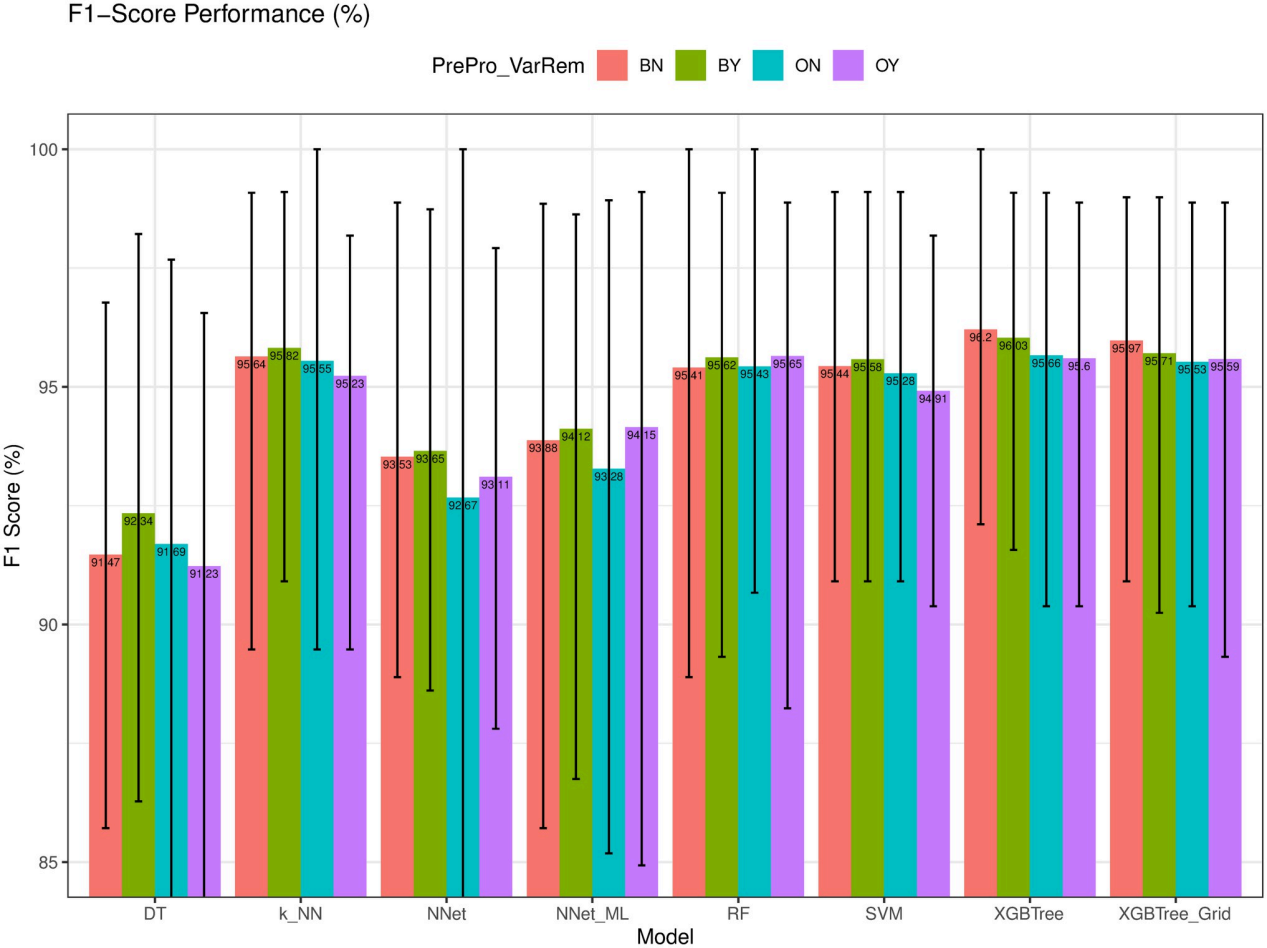
F1-score (*F1-score*) of different models.

We compared the Precision (*Pre*) and F1-score (*F1-score*) of different models in Figs 12 and 13, respectively. As observed, *k-NN* had the highest *Spe* of 95.65%, whereas *XGBTree* achieved the highest *F1-score* = 96.20%. The high value of *Pre* of *k-NN* was possibly due to the dominance of the benign cases which were the majority class in the dataset.

## Discussion

### Identifying the best model

Table 5 shows the best model by different evaluation metrics. The values in the table were the average of all the runs by the same model for a specific configuration. We note that the values in Table 5 are different from the values in Table 3 because the values in Table 3 are the average of all runs across all configurations of the same model. Our experiment results showed that *XGBTree* trained with ENUS achieved the best performance in most of the evaluation measures including accuracy (*Acc*), balanced accuracy (*bAcc*), sensitivity (*Sen*), and the F1 score (*F1-Score*). We will further evaluate the efficacy of ENUS with different imbalanced datasets in our next subsection.

**Table 5:** XGBTree achieved best performance in most of the evaluation metrics (PrePro—Pre-processing type (B—Balanced (ENUS), O—Original data); VarRem—Variable removal (Y—Yes, N—No)).

Metric	Best Model	PrePro	VarRem	Avg. Value
*Acc*	XGBTree	B	N	97.36%
*bAcc*	XGBTree	B	N	97.47%
*Sen*	XGBTree	B	N	97.88%
*Spe*	k-NN	O	N	97.68%
*Pre*	k-NN	O	N	95.65%
*F1-Score*	XGBTree	B	N	96.20%

### Identifying feature importance

**Fig 14:**
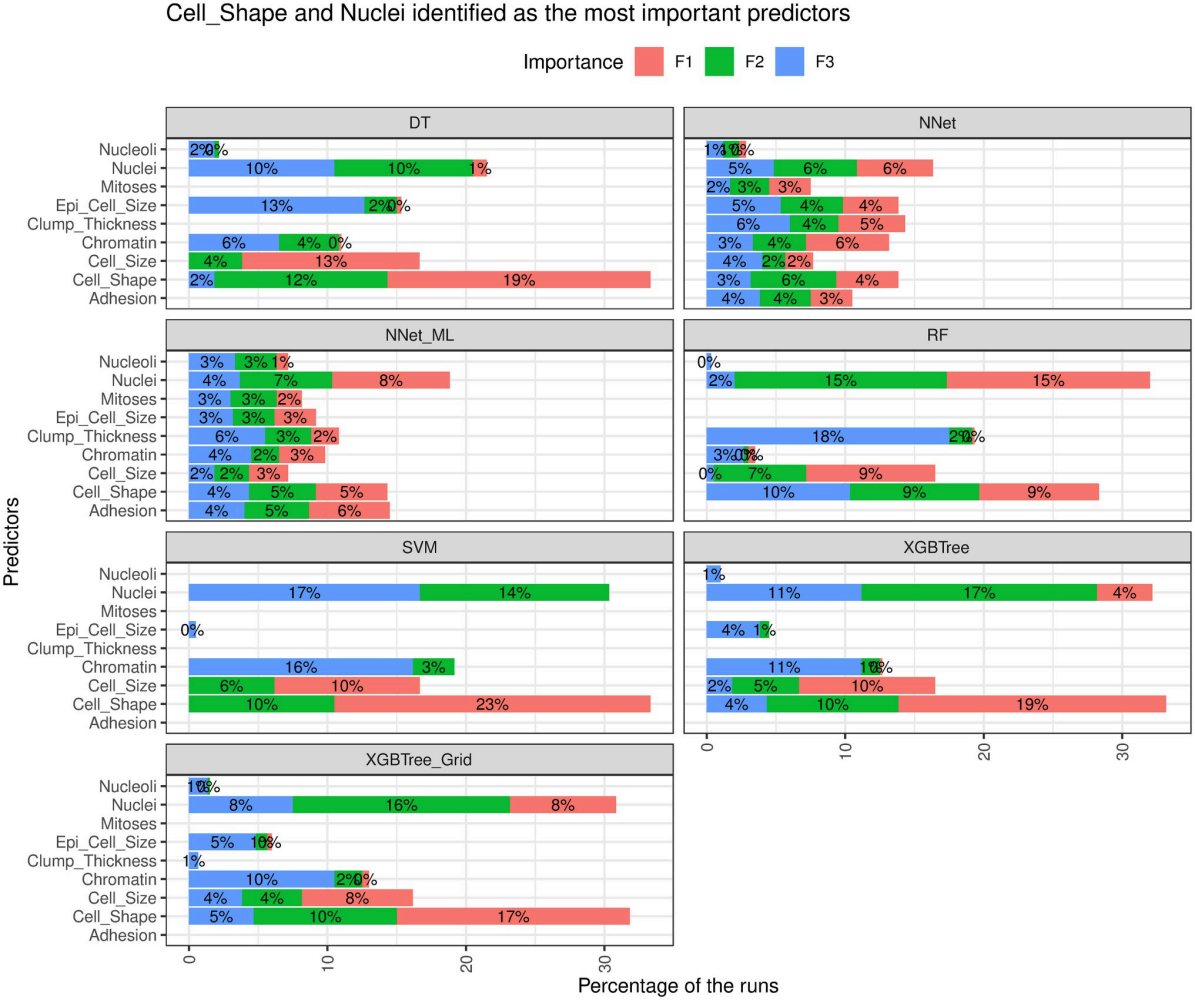
*Cell_Shape* and *Nuclei* were identified as the most important predictors.

Next, we analyzed the models to identify the most important features (IFs) in predicting the target variable. In our analysis, the IFs identified by different training configurations were combined for each model. The feature importance is calculated based on the models. For example, to determine the feature importance in a decision tree (DT) model, we calculated the mean decrease in impurity for each feature across all trees and the feature with the most impurity deduction was marked as the most importance. Fig 14 shows the feature importance identified by each model, where F1, F2 and F3 indicate the first, second and third important features, respectively. Because the *k-NN* model used all features and optimized the prediction accuracy based on the number of neighbors, all predictors were considered with the same level of importance in *k-NN*; thus, the model was removed from the analysis. As expected, the key features identified by the models were similar. As shown in the plot, *Cell_Shape* and *Nuclei* were identified as the two most important features in predicting breast cancer risk in all models. For example, in 34% of the runs *DT* identified *Cell_Shape* is in the top three of the most important features, whereas in 30% of the runs *Nuclei* was identified as one of the top 3 most important features in predicting breast cancer. This is consistent with the knowledge in the biology literature of *Cell_Shape* being strongly associated with benign and malignant breast cancer cases [44]. In *XGBTree*, *Nuclei* was identified as the most important predictor, followed by *Cell_Shape*, and *Chromatin*. The finding is interesting as it reaffirmed the reported correlation between nuclear morphometry and the grades of breast cancer [45, 46]. Instead of employing the tedious task of using cell images linked with the grade levels of breast cancer, our machine learning model based approach utilize the data-driven to characterize the underlying relationships between the predictors and target variable to achieve the same result. It’s also interesting to observe that the neural network models considered all predictors with the same importance, and ended up with the worst performance. This is counterintuitive to our data exploration analysis as we observed that some of the predictors would not perform well in separating the classes of the target variable. For example, based on our exploratory analysis, *Clump_Thickness* should be considered as the least important feature in predicting breast cancer. This is consistent with the feature importance outcome shown in Fig 14.

### Training runtime evaluation

**Fig 15:**
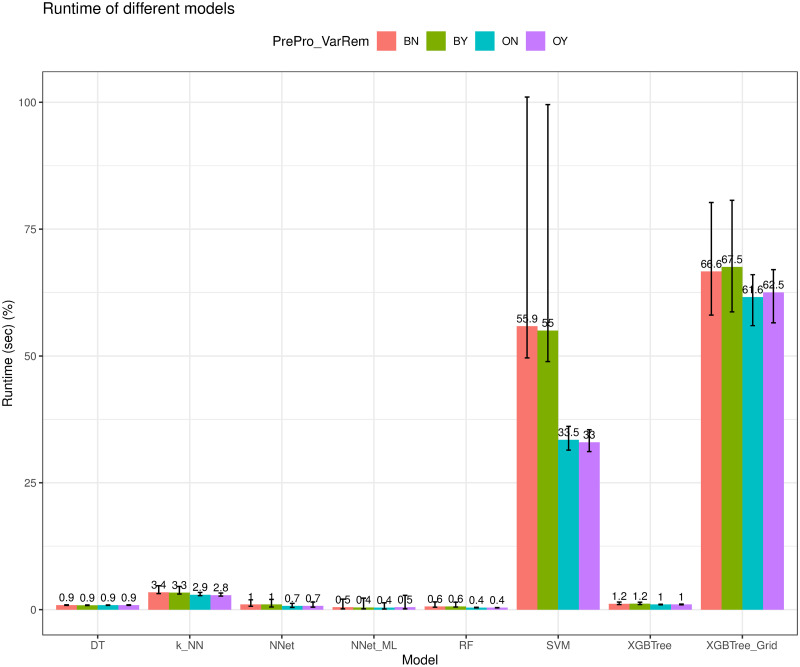
Average runtime for training different models.

We next evaluate the training time of different models. In our experiment, we ran the program using *R* programming language version 4.0.2 (2020-06-22) on the Windows 10 x64 operating system (build 19043, 32GB RAM). The runtime of a model is measured by the time difference between the start time and the end time for training a model using the *Sys.time()* function in R. We ran 100 trials and computed the average of the runtimes of different models. We note that during the runtime evaluation, there were no other programs running. Fig 15 shows the average runtime on training different models with the error bar representing the minimum and maximum of the runtimes. As we observed, *NNet-ML* had the best rutime, on the order of fractions of a second, thanks to the training algorithm of the *neuralnet* package [38]. We also observed that the other models that did not use the grid search algorithm also had very short training runtime in the order of seconds. On the other hand, *XGBTree-Grid* and *SVM* had the worst training runtime due to the use of the grid search algorithm to find the best hyperparameters during the model training process. As we discussed above, the prediction accuracy performance gains were not significant; thus it is not worth using the grid-search algorithm in this case.

### Model evaluation with different imbalanced datasets

We also tested the proposed approach ENUS with different datasets having different ratios between the minority and majority classes. To get a dataset that has a specific ratio of the minority to majority classes, we used the original dataset and randomly selected numbers of examples in the two classes to meet the ratio requirement. For example, to have a dataset where ratio of minority class to the majority class equals 5%, we select all the majority class examples (444) while randomly select only 23 examples of the minority class from the original dataset. We then randomly divide the newly generated dataset into training (80%) and validation (20%) datasets while still maintaining the minority to majority class ratio in these subsets. The training dataset is used for training the models while the evaluation dataset is used for model evaluation. The training and evaluation procedure is the same as that of the original dataset shown in Fig 7. In our experiment, we changed the ratio between the minority class to the majority class from 5% to 50%.

**Fig 16:**
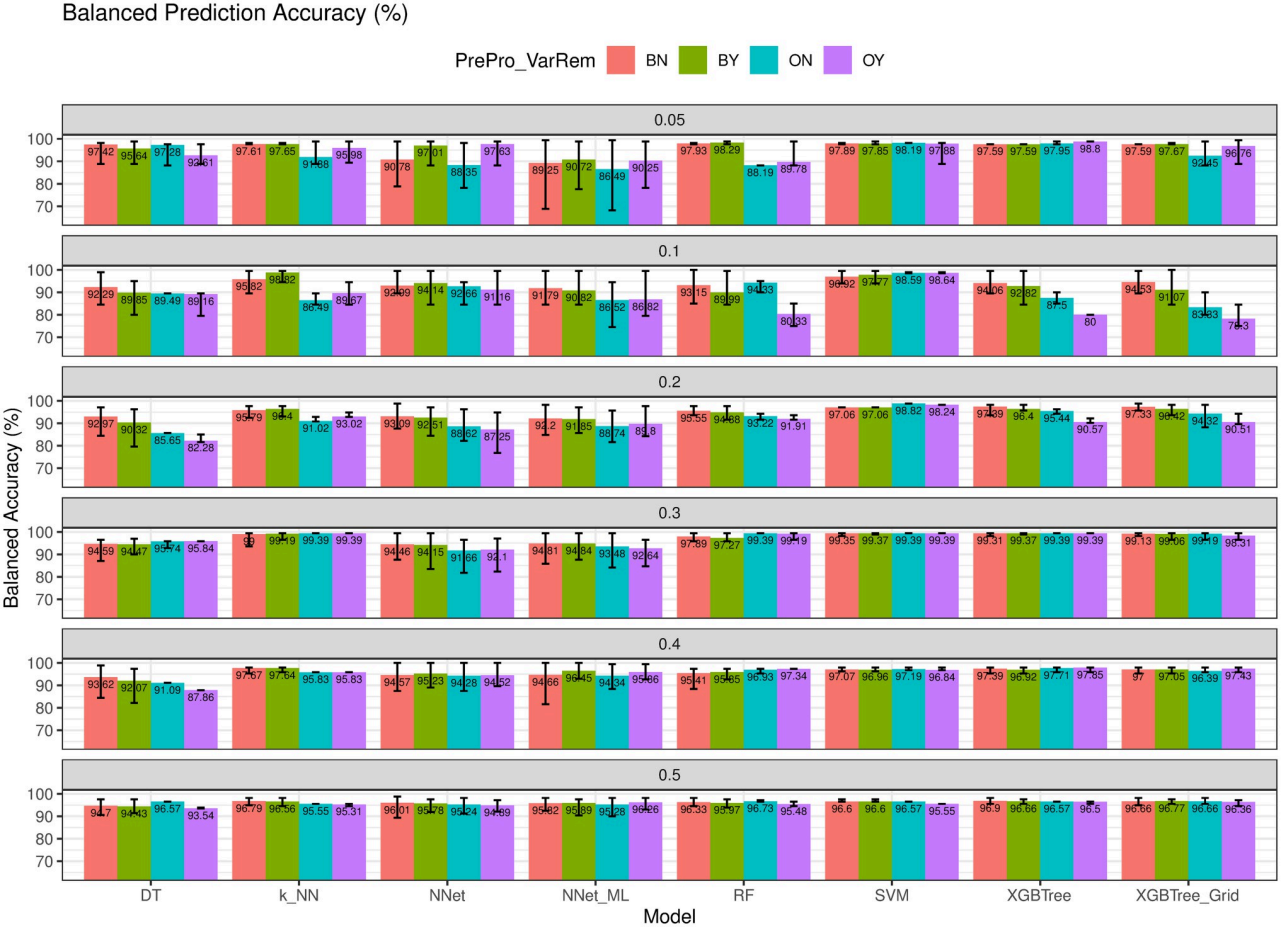
Balanced accuracy with different ratios of minority to majority classes. Models trained with ENUS improved by 2.04% compared with the models trained with the original data.

Using imbalanced datasets, we are interested in the performance of the models on the balanced accuracy, sensitivity and F1-score because the malignant cases are rare and much less than that of the benign cases. Also, correctly classifying these cases is important from the application aspects. The results of the experiments on the balanced accuracy (bAcc) are illustrated in Fig 16. Overall, the models trained with ENUS improved their performance by 2.04% compared to those trained with the original data. The dataset with the ratio of minority to the majority class of 10% had the highest improvement of 5.24% on balanced accuracy.

**Fig 17:**
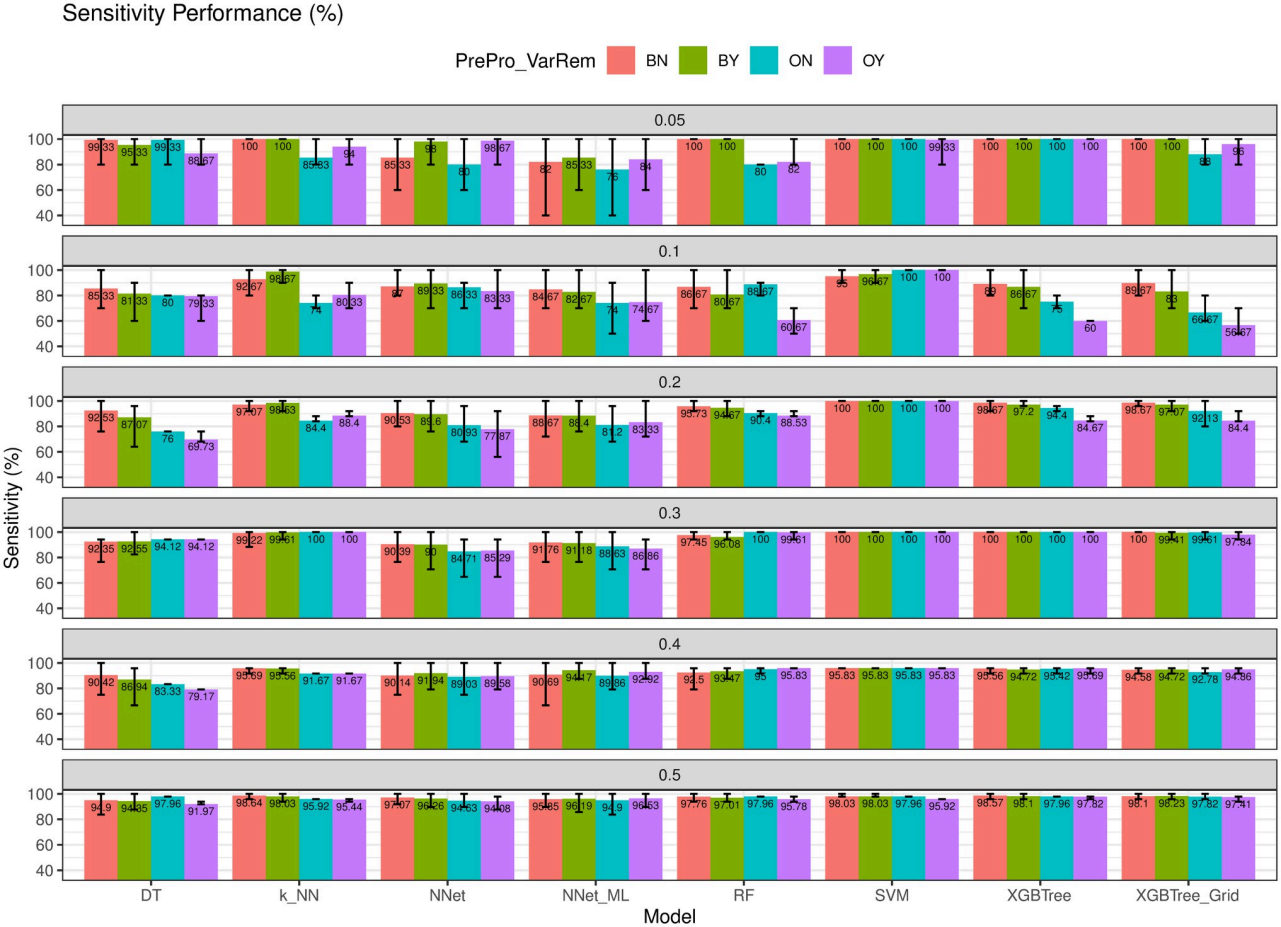
Sensitivity with different ratios of minority to majority classes. Models trained with ENUS improved the performance by 4.69%.

Fig 17 shows the sensitivity performance of the models in different datasets. We observed that on average the models trained with ENUS improved the sensitivity by 10.6% compared to those used the original data in the 10% dataset (i.e., the minority cases are 10% of the majority cases). We also observed that in some model, the improvement can reach up to more than 15% when the ratio is small. For instance, the RF models trained with ENUS improved 19% compared to the RF models trained with the original data in the 5% dataset. On average, balancing the training data improved the sensitivity performance of the models by 4.69%.

**Fig 18:**
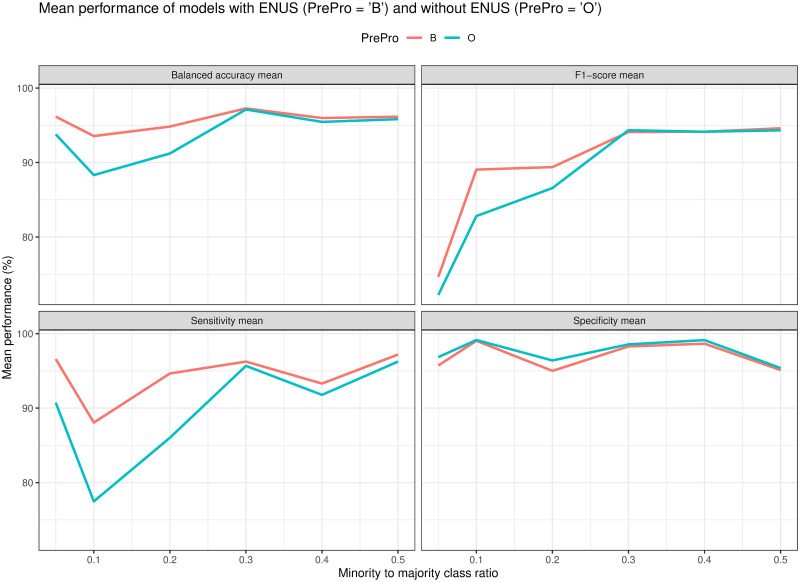
Mean performance of all models with different ratios of minority to majority classes. Models trained with balanced training data (PrePro = ‘B’) significantly improved compared to those trained with original data (PrePro = ‘O’), especially in the smaller range of the ratios.

Finally, Fig 18 shows the performance of the models change when we vary the ratio of the minority to the majority classes. In the experiment, we used the mean performance of all models using ENUS (PrePro = ‘B’) and not using ENUS (PrePro = ‘O’). We first observed that the specificity of the models with and without ENUS are quite similar. This is because ENUS has less impact on the majority class as there were plenty of its records in the evaluation dataset. However, the models using ENUS outperformed the others without using it in balanced accuracy, sensitivity, and F1 score, especially, in the smaller range of the ratios of the minority to majority classes. When the ratio of the minority class to the majority class is less than 20%, on average, the models with ENUS improved 3.74% on balanced accuracy, 8.36% on sensitivity, and 3.83% on F1 score.

### Computational complexity analysis

We assume that *n* is the number of examples of the minority class that we need to generate. Using the traditional up-sampling algorithm, examples of the training dataset are randomly selected and duplicated. The process is repeated *n* time; thus, the runtime complexity of the traditional algorithm is *O*(*n*). On the other hand, using ENUS, to generate a new example, we need to randomly select an example (i.e., a Centroid) from the training dataset and find *k* ≤ *m* nearest neighbors of it (*m* is the number of minority examples in the training dataset). Assume that *d* is the dimensions of the features of the dataset; thus, the runtime complexity for calculating the distances is *O*(*d* × *m*). Once the distances are calculated, we then order them to find the least *k* distances. Using the merge sort algorithm [47], its runtime is *O*(*m* × *log*(*m*)). The process is repeated *n* times; thus, the computational complexity of ENUS is *O*(*n* × *d* × *m*^2^ *log*(*m*)). As expected, ENUS has higher computational complexity compared to the duplication approach. However, its runtime is still polynomial time and the values of *m* and *d* are typically very small; thus, the computational overhead of ENUS compared to the traditional duplication approach is negligible.

### Major findings

This study investigates machine learning-based methods to classify breast cancer patients based on the preliminary diagnostic data. The proposed approach is very effective and accurate with an average prediction accuracy of 97.36% (min = 93%, max = 100%), balanced accuracy of 97.47% (min = 93%, max = 100%), and sensitivity of 97.88% (min = 89%, max = 100%). Our proposed approach ENUS for handling imbalanced training data achieved higher performance compared to the models that did not. The models trained with ENUS significantly improved the balanced accuracy by 2.4% and sensitivity by 4.46% compared to those trained with original data. In the range of smaller ratio between the minority to majority class (e.g., less than 20%), the improvement is larger with 3.74% balanced accuracy and 8.36% sensitivity. In addition, we identified the key attributes that play important roles in classifying benign versus malignant patients. Particularly, our study revealed that *Cell_Shape* and *Nuclei* were the most important predictors in classifying breast cancer cases. Our finding re-affirmed the correlation between nuclei morphometry and the grades of breast cancers that have been reported in other studies [44, 45]. Furthermore, we also quantified and compared the training effectiveness of different models. Our study can be readily integrated into the existing healthcare systems to help healthcare professionals to more precisely and effectively identify and treat the patients. To the best of our knowledge, this is one of a few comprehensive studies comparing a wide range of advanced machine learning models in predicting breast cancer risk using a small-size clinical dataset.

### Limitations

The study had some limitations regarding the small size of the dataset. We had a total of 682 complete cases. Our model would likely have achieved higher performance if more data were available in the training process. Another limitation was the number of predictors in the dataset (10 predictors). The machine learning based methods that can search for the patterns in large datasets and select the best set of predictors for classification can be fully utilized for higher performance with more predictors.

## Conclusion

Our study provided a novel approach for handling imbalanced data to improve the predictive performance of machine learning-based models using a small-size dataset. Our experimental results showed that the models trained with the proposed ENUS approach improved the prediction balanced accuracy by 2.4% and the sensitivity by 4.69% compared to those using original data for training. Particularly, in the range of smaller ratio between the minority to majority class (e.g., less than 20%), the improvement is much larger with 3.74% on the balanced accuracy and 8.36% on the sensitivity. *XGBTree* model trained with ENUS achieved the best performance with an average classification accuracy of 97.36% (max = 100%, min = 93%). The performance improvement was validated using the independent *t*-test with a 95% confidence interval. Additionally, our study revealed that *Cell_Shape* and *Nuclei* were the most important predictors in classifying breast cancer. Finally, we measured the training time to compare the effectiveness of different models. Our proposed approach can be used by healthcare professionals for early detection of breast cancer, which would support more effective treatment of patients. For future study, a similar dataset with additional demographics or genetic information will be used to construct the models. Fusing information with different modalities is challenging but it’s worth exploring as we expect it will improve the model performance.
